# Pathogenic variants of Alport syndrome and monogenic diabetes identified by exome sequencing in a family

**DOI:** 10.1038/s41439-023-00233-0

**Published:** 2023-02-02

**Authors:** Hirofumi Watanabe, Shin Goto, Michihiro Hosojima, Hideyuki Kabasawa, Naofumi Imai, Yumi Ito, Ichiei Narita

**Affiliations:** 1 Division of Clinical Nephrology and Rheumatology, Kidney Research Center, Niigata University Graduate School of Medical and Dental Sciences, Niigata, Japan; 2 Department of Clinical Nutrition Science, Kidney Research Center, Niigata University Graduate School of Medical and Dental Sciences, Niigata, Japan; 3 Department of Health Promotion Medicine, Niigata University Graduate School of Medical and Dental Sciences, Niigata, Japan

**Keywords:** Disease genetics, Kidney diseases, Diabetes

## Abstract

We present a family of two female Alport syndrome patients with a family history of impaired glucose tolerance. Whole exome sequencing identified a novel heterozygous variant of *COL4A5* NM_033380.3: c.2636 C > A (p.S879*) and a rare variant of *GCK* NM_001354800.1: c.1135 G > A (p.A379T) as the causes of Alport syndrome and monogenic diabetes, respectively. Two independent pathogenic variants affected the clinical phenotypes. Clinical next-generation sequencing is helpful for identifying the causes of patients’ manifestations.

Clinical next-generation sequencing is increasingly being used in medical practice to diagnose diseases^1^. However, the determination of the causative pathogenic mutations in cases with multiple disease phenotypes remains challenging. Here, we report a family that presented with two independent hereditary diseases, Alport syndrome by a novel pathogenic variant and monogenic diabetes by a rare pathogenic variant, that was identified by whole exome sequencing.

Alport syndrome is a genetically and phenotypically heterogeneous disorder of glomerular, cochlear, and ocular basement membranes that result from mutations in the genes of the α3, α4, and α5 chains of collagen type IV^2^. The major gene that causes Alport syndrome is *COL4A5*, which encodes the collagen IV α5 chain, with X-linked inheritance^3^. Due to the phenotypic heterogeneity in heterozygous females^4^, genetic analysis is useful for diagnosing X-linked Alport syndrome in females.

Diabetes mellitus is a common disease with heterogeneous clinical phenotypes. Although many factors affect the phenotypes of diabetes, small populations of diabetes patients show monogenic forms^5^. In these monogenic diseases, genetic testing can identify the etiological subtypes, which have profound implications on treatment, and can predict the future development of associated clinical features, allowing early preventative or supportive treatments^6^.

In the present study, the proband was a 36-year-old female. Initially, she presented with microscopic hematuria at 6 years of age and received her first renal biopsy at 7 years of age. She was diagnosed with glomerulonephritis. Due to persistent hematuria and proteinuria, she received a second renal biopsy at 13 years of age and was diagnosed with glomerulonephritis of unclassified pathology. After the second biopsy, she received corticosteroid therapy. However, remission of the urine abnormality was never achieved. Later, she was diagnosed with gestational diabetes mellitus during her first pregnancy when she was 28 years old. The patient received a third renal biopsy when her first daughter showed a urine abnormality, and a hereditary renal disease was suspected (Fig. 1A). At the time of the renal biopsy, her renal function was normal, with a serum creatinine level of 0.62 mg/dL and creatinine clearance of 93 mL/min/1.73 m^2^. Her urine protein level was 0.5 g/day, and urine sediment examination revealed hematuria with 20 to 29 red blood cells/high-power field. Under light microscopy, the glomeruli showed only mild focal segmental proliferation of the mesangium, and none of the findings were indicative of any specific renal disease. Immunofluorescence studies did not show the deposition of any immunoglobulin. However, electron microscopy demonstrated reticular changes in the glomerular basement membrane (Fig. 2A), suggesting Alport syndrome. Immunostaining for collagen type IV revealed the pattern for X-linked Alport syndrome in females; the intensity of the collagen IV α5 chain was decreased in some glomerular walls (Fig. 2B). Therefore, the patient was histologically diagnosed with Alport syndrome. Her first daughter, who had similar renal biopsy findings (Fig. 2C) and showed mild glucose intolerance at the time of the renal biopsy, was also diagnosed with Alport syndrome. Neither patient had ever shown any hearing loss or ocular abnormalities. No other family member demonstrated urinary, hearing, or visual abnormalities. However, there was a family history of impaired glucose tolerance that spanned four generations (Fig. 1A). In the proband, the hemoglobin A1C level was 6.4%, and the diabetes was not severe in the rest of the family members with hyperglycemia.

**Fig. 1:**
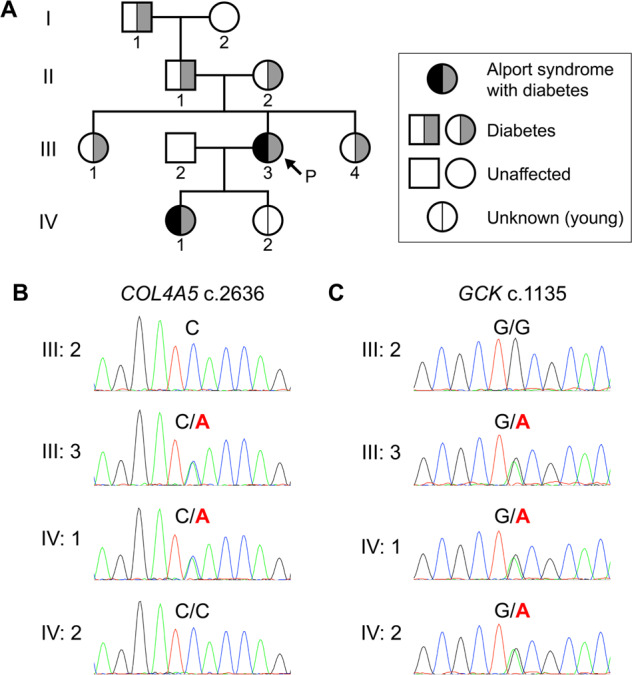
The familial pedigree and genetic analysis. **A** The family tree. Square symbols indicate males, and round symbols indicate females. The arrow indicates the proband. **B** Sanger sequencing for *COL4A5*. **C** Sanger sequencing for *GCK*. Green, blue, black, and red lines represent adenine (A), cytosine (C), guanine (G), and thymine (T), respectively.

**Fig. 2:**
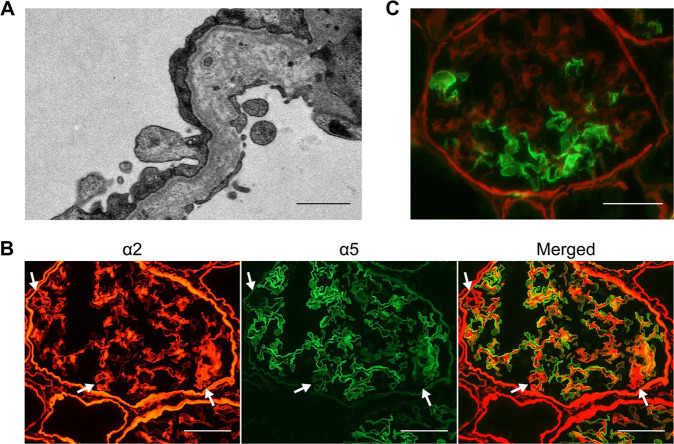
Renal histopathological findings of the patients with Alport syndrome. **A** Electron microscopy of the glomerular basement membrane in the proband (III:3). Bar = 1 µm. **B** Immunofluorescence staining of the type IV collagen α2 chain (red) and α5 chain (green) in the renal biopsy sample of the proband (III:3). Normal diffuse staining of the α2 chain was seen in the glomerular basement membrane, Bowman’s capsule, and tubular basement membrane, whereas discontinuous staining of the α5 chain was seen. Arrows indicate regions where the α5 chain was not well expressed when compared to the α2 chain. Bar = 50 µm. **C** Immunofluorescence staining of the type IV collagen α2 chain (red) and α5 chain (green) in the renal biopsy sample of the proband’s first daughter (IV:1). Bar = 50 µm.

We performed whole exome sequencing with blood samples from four members of the family, i.e., the proband (III:3), the proband’s spouse (III:2), the proband’s first daughter (IV:1), and the proband’s second daughter (IV:2; Fig. 1A). Informed consent and assent were obtained from all participants or their parents. This study was approved by the Institutional Review Board of Niigata University Graduate School of Medical and Dental Sciences, Niigata, Japan (approval no. G2021-0035) and was carried out according to the principles of the Declaration of Helsinki. We explored rare variants that were not registered in dbSNP^7^. Of these, we detected 11 variants only in the patients with Alport syndrome (III:3 and IV:1; Supplementary Table S1). Six variants that were unregistered in dbSNP were detected only in the patients (III:3 and IV:1) and the proband’s second daughter (IV:2), whose phenotype was unknown because she was very young at the time of genome sampling (Supplementary Table S2). Based on the clinical phenotypes, the predicted influence on proteins, and considerations from the literature, we identified a novel heterozygous variant of *COL4A5* NM_033380.3: c.2636 C > A (p.S879*) and a rare heterozygous variant of *GCK* NM_001354800.1: c.1135 G > A (p.A379T) as the causes of Alport syndrome and diabetes, respectively. We confirmed these variants by Sanger sequencing (Fig. 1B, C). *COL4A5* c.2636 C > A was classified as likely pathogenic (PVS1, and PM2), and *GCK* c.1135 G > A was classified as pathogenic (PM1, PM5, PP3, and PM2) according to the American College of Medical Genetics and Genomics (ACMG) guidelines^8^.

*COL4A5* is very large, encompassing 250 kb of genomic DNA with 51 exons encoding a 6.5-kb transcript^9^, and many variants in *COL4A5* have been identified to be causative variants of X-linked Alport syndrome. In this study, we identified a novel heterozygous nonsense mutation in *COL4A5* in two female patients. Strong genotype-phenotype correlations have been identified in male X-linked Alport syndrome, and patients with truncating mutations due to nonsense mutations, small insertions, or deletions that lead to a premature stop codon show severe phenotypes^10^. The p.S879* in *COL4A5* that we detected in this study is expected to have a severe influence on the phenotype. Heterozygous X chromosome mutations induce heterogeneous phenotypes. In the present study, the renal biopsy results showed that the proband (III:3) had a milder collagen IV α5 chain defect than her first daughter (IV:1).

*GCK* encodes glucokinase, an enzyme that catalyzes glucose phosphorylation at the initiation of glycolysis; defects of this gene change the glucose-stimulated insulin secretion threshold^11^. Hyperglycemia related to *GCK* is the most common cause of monogenic diabetes^5^. However, *GCK* c.1135 G > A (p.A379T) is a rare variant; it was reported only in a recent paper as a novel pathogenic variant^12^. It has been reported that A379 is located at the back of the ATP-binding site and that A379T changes the enzymatic activity of glucokinase^13^. In addition, variants at the same amino acid site (c.1136 C > T (p.A379V^14^) and c.1136 C > A (p.A379E^15^)) have been reported as causative mutations of monogenic diabetes. Generally, hyperglycemia due to mutations of *GCK* is much milder than other types of monogenic diabetes and is thus frequently underdiagnosed, although it is sometimes found during pregnancy^16^. This is consistent with the mild diabetic phenotype seen in the family described in the present study. In this family, both of the proband’s parents (II:1 and II:2) presented diabetes. One of them could have the *GCK* c.1135 G > A, and another should not have the variant and may have a different cause for diabetes. Because of the unavailability of the genome samples, we could not completely verify that the *GCK* c.1135 G > A affected hyperglycemia for all four generations. Hyperglycemia due to *GCK* mutations rarely requires pharmacological treatment, and it rarely induces diabetes-related complications^17^. The identification of mutations by genomic testing may be useful in allowing avoidance of unnecessary treatments in those with hyperglycemia related to *GCK*.

Predicted pathogenic *COL4A5* variants are estimated to be present in at least 1 in 2320 individuals^18^, although the exact prevalence of X-linked Alport syndrome is unknown. The estimated incidence of monogenic diabetes due to *GCK* mutations is 1 in 1000 individuals^19^. Hence, the combination of these two diseases should not be extremely rare. However, to our knowledge, this is the first report to identify genetic mutations of Alport syndrome and monogenic diabetes in the same family.

In conclusion, we found a novel variant in *COL4A5* and a rare variant in *GCK* in a family that presented with Alport syndrome and monogenic diabetes. Two independent rare pathogenic variants affected the clinical phenotypes. Clinical next-generation sequencing is helpful for identifying the causes of patients’ manifestations.

## HGV Database

The relevant data from this Data Report are hosted at the Human Genome Variation Database at 10.6084/m9.figshare.hgv.3267, 10.6084/m9.figshare.hgv.3270.

### Supplementary information

Supplementary Tables
