# Supplementary material for: Pathogenic variants of Alport syndrome and monogenic diabetes identified by exome sequencing in a family
Source: Hum Genome Var. 2023 Feb 2;10:5. doi: 10.1038/s41439-023-00233-0 (PMC9894847; doi:10.1038/s41439-023-00233-0)
Supplement: Supplementary file 1 — Supplementary Tables [file 41439_2023_233_MOESM1_ESM.docx]

**Supplementary Table S1. Variants unregistered in dbSNP detected in III:3 and IV:1.**

| Chromosome | Position | Reference | Alternative | Gene | cDNA | Protein |
| --- | --- | --- | --- | --- | --- | --- |
| 1 | 156170170 | G | T | *SLC25A44* | c.532G>T | p.G178* |
| 2 | 175330609 | T | C | *GPR155* | c.1288A>G | p.M430V |
| 4 | 95561523 | A | G | *PDLIM5* | c.1292A>G | p.D431G |
| 10 | 25279474 | TCT | - | *ENKUR* | c.510_512del | p.E170del |
| 14 | 24470614 | A | G | *DHRS4L2* | c.553A>G | p.S185G |
| 15 | 40705230 | T | C | *IVD* | c.737T>C | p.L246P |
| 16 | 88501044 | G | A | *ZNF469* | c.7166G>A | p.G2389D |
| 17 | 9489122 | G | A | *CFAP52* | c.103G>A | p.D35N |
| 17 | 68129151 | A | G | *KCNJ16* | c.923A>G | p.H308R |
| 20 | 402847 | T | A | *RBCK1* | c.994T>A | p.C332S |
| **X** | **107863615** | **C** | **A** | ***COL4A5*** | **c.2636C>A** | **p.S879*** |

**Supplementary Table S2. Variants unregistered in dbSNP detected in III:3, IV:1, and IV:2.**

| Chromosome | Position | Reference | Alternative | Gene | cDNA | Protein |
| --- | --- | --- | --- | --- | --- | --- |
| 2 | 110372959 | A | T | *SOWAHC* | c.893A>T | p.D298V |
| 6 | 134350598 | A | C | *SLC2A12* | c.365T>G | p.L122* |
| 7 | 36373574 | C | - | *KIAA0895* | c.1197del | p.M399Ifs*60 |
| **7** | **44185214** | **C** | **T** | ***GCK*** | **c.1135G>A** | **p.A379T** |
| 10 | 88702802 | G | A | *MMRN2* | c.1739C>T | p.A580V |
| 19 | 18724952 | G | T | *TMEM59L* | c.355G>T | p.A119S |

**Supplementary Table S3. Primers for Sanger sequencing.**

| Gene | Primer | Sequence (5' to 3') |
| --- | --- | --- |
| *COL4A5* | Forward | AGGTCTGTTATCTACAGGGTTC |
|  | Reverse | CAAAGTGAGCTTCCTGCAGTC |
| *GCK* | Forward | GACCGCAAGCAGATCTACAACA |
|  | Reverse | AAGGGGGACGAGAAGAGGACTA |
